# Accuracy of yellow fever case definition of epidemiologic surveillance, São Paulo, 2018

**DOI:** 10.11606/s1518-8787.2023057005001

**Published:** 2023-07-18

**Authors:** Ana Freitas Ribeiro, Roberta Figueiredo Cavalin, Aparecida Mei Mingrone Klimas, Ricardo Manfredo, Luciana Marques Sansão Borges

**Affiliations:** I Secretaria de Estado da Saúde de São Paulo Instituto de Infectologia Emílio Ribas São Paulo SP Brasil Secretaria de Estado da Saúde de São Paulo. Instituto de Infectologia Emílio Ribas. São Paulo, SP, Brasil; II Universidade Municipal de São Caetano do Sul Faculdade de Medicina São Paulo SP Brasil Universidade Municipal de São Caetano do Sul. Faculdade de Medicina. São Paulo, SP, Brasil

**Keywords:** Yellow Fever, Accuracy, Case Definition, Epidemiologic Surveillance

## Abstract

**OBJECTIVE:**

To evaluate the accuracy of yellow fever (YF) suspected case definitions from the Brazilian Ministry of Health (BMH) and World Health Organization (WHO), as well as propose and evaluate new definitions of suspected cases, considering confirmed and discarded cases.

**METHODS:**

The retrospective study was conducted at the Instituto de Infectologia Emílio Ribas (IIER), using the Epidemiologic Surveillance Form of YF cases. From the confirmed and discarded cases of YF, a logistic regression model was developed. The independent variables were used in a proposed definition of a suspected case of YF and its accuracy was evaluated.

**RESULTS:**

In total, 113 YF suspect cases were reported, with 78 confirmed (69.0%). The definitions by BMH and WHO presented low sensitivity, 59% and 53.8%, and reduced accuracy, 53.1% and 47.8%, respectively. Predictive factors for YF were thrombocytopenia, leukopenia, and elevation of transaminases greater than twice normal. The definition including individual with acute onset of fever, followed by elevation of ALT or AST greater than twice the reference value AND leukopenia OR thrombocytopenia presented high sensitivity (88.3%), specificity (62.9%), and the best accuracy (80.4%), as proposed in the model.

**CONCLUSION:**

The YF suspected case definitions of the BMH and the WHO have low sensitivity. The inclusion of nonspecific laboratory tests increases the accuracy of YF definition.

## INTRODUCTION

Yellow fever (YF) is an acute disease, endemic in some tropical areas of the Americas and Africa, and it can affect humans and non-human primates (NHP). It is caused by a virus of the genus *Flavivirus*, family *Flaviviridae*, and transmitted by mosquitoes. There are two cycles of transmission: sylvatic and urban. YF is a burden for public health due to its clinical severity and high potential for dissemination in urban areas^1^. Vaccination is the most important measure to prevent YF, especially its international spread. The World Health Organization (WHO) developed, by a coalition of partners (The Vaccine Alliance-GAVI and UNICEF) the comprehensive global strategy to eliminate yellow fever epidemics (EYE) (2017–2026), to face the changing epidemiology of yellow fever, resurgence of mosquitoes and increased risk of urban outbreaks and international spread^2^. The last case of urban yellow fever in Brazil occurred in 1942, despite the sporadic record of cases of sylvatic YF. The disease was endemic until 1999, especially in the North and Central-West regions. Between 2000 and 2008, an expansion of a viral circulation was observed towards the East and South regions^3^.

From December 2016 to May 2017, 792 cases of sylvatic YF were reported and 274 deaths were confirmed (lethality 34.5%), predominating in the states of Minas Gerais (61.4%) and Espírito Santo (32.8%), with also 642 confirmations in NHP^4^. From July 2017 to April 2018, 1,127 human cases of sylvatic YF and 331 deaths were confirmed (lethality 29.4%), predominating in the states of Minas Gerais (42.6%), São Paulo (40.2%) and Rio de Janeiro (16.6%)^5^.

YF is a notifiable disease in Brazil. Its definition of a suspect case is an individual with fever (up to 7 days) of sudden occurrence followed by jaundice and/or hemorrhagic manifestations, residing in (or coming from) a YF risk area, or in locations with confirmed epizootic in NHP or of isolation of virus in vector mosquitoes in the 15 days prior, not vaccinated against YF, or with ignored vaccine status^6^. In 2009, a study analyzed 28 confirmed cases of sylvatic YF in the State of São Paulo. However, only 50% of the cases matched the definition of suspect case^7^.

This study aimed to describe the suspect cases attended at the Instituto de Infectologia Emílio Ribas (IIER), to evaluate sensitivity and specificity of YF case definitions from the Brazilian Ministry of Health and WHO during the 2018 outbreak, and to propose highly accurate case definitions.

## METHODS

### Study Design: Retrospective Observational Study

Data sources: data from patients with suspected YF treated at the IIER were collected using the Epidemiological Surveillance Forms, selecting sociodemographic, clinical, and laboratory variables. Most patients were from the State of São Paulo (96.2%), mostly from the municipality of Mairiporã (41.0%).

Inclusion requirement: suspect cases had a clinical sample collected for laboratorial etiological confirmation provided by Instituto Adolfo Lutz, Public Health Laboratory. Suspected cases of YF were confirmed or discarded by YF virus detection by real-time polymerase chain reaction (RT-PCR) technique (91% and 91.4%) or positive immunoglobulin M (IgM) serology (8% and 5.7%), respectively.

Dependent variable: case classification (confirmed or discarded)

Independent variables: sociodemographic, clinical (signs and symptoms), and laboratory tests.

### Analysis Procedure

Characteristics and the confirmed and discarded cases for YF were evaluated by Pearson’s chi-squared test and Fisher’s exact test. Univariate analysis of signs, symptoms, and laboratorial alterations of suspect cases were performed in order to identify possible variables predictive of YF, considering odds ratio (OR) values. The variables with p-value ≤ 0.20 in non-adjusted analysis were considered for a logistic regression model to identify independent factors associated to YF. The final models were evaluated by Hosmer-Lemeshow test.

Receiver operating characteristic (ROC) curves were constructed for each of the explanatory models, and the predictive factors were considered to create proposed definitions (1 and 2). Those proposed definitions were evaluated by sensitivity, specificity, predictive value positive (PVP), predictive value negative (PVN), and accuracy, which were also used to evaluate the following official definitions of suspect case.

### Brazil’s Ministry of Health’s – Brazil’s definition (2017)

An individual with acute onset of fever (up to 7 days), followed by jaundice and/or hemorrhagic manifestations, residing in (or coming from) a risk area for YF, or from locations with occurrence of epizootic confirmed in NHP, or isolation of virus in vector mosquitoes, in the 15 days prior, not vaccinated against YF, or with ignored vaccine status. In cases of outbreak, it is recommended to adjust the definition of suspect case making it more sensitive for detection of the highest number of cases possible, taking in consideration the wide clinical spectrum of the disease^6^.

### World Health Organization’s – WHO definition (2015)

Any individual with acute onset of fever and jaundice occurrence within 14 days of onset of the first symptoms^8^.

For the analysis of both definitions (Brazil and WHO), a vaccinated individual was considered as one with immunization received in, at least, 10 days from the beginning of the symptoms^9^. Moreover, the cases with non-reported traveling to or residence in YF risk areas were included as possible exposition to sylvatic environments during the YF outbreak. Laboratorial value patterns considered to describe observed alterations were the following: thrombocytopenia with platelet count equal or under 150,000/mm3; leukopenia with leukocyte count equal or under 4,500/mm3; leukocytosis with leukocyte count equal or over 11,000/mm3; renal function alterations with serum urea values over 40mg/dL and/or creatinine over 1.3 mg/dL; hyperbilirubinemia with total bilirubin serum dosage over 2 mg/dL; and increase of twice the reference value for transaminases considering the maximum value of 40 U/L for alanine aminotransferase (ALT) and aspartate aminotransferase (AST).The significance level adopted was 5% for all hypothesis tests. Analyses were performed using SPSS for Windows v.25 and Stata/MP 14.0 for Windows software. The study used data from hospital epidemiological surveillance. The data was approved by the Ethics Committee of IIER (Protocol 024329/2018).

## RESULTS

In total, 113 suspect cases of YF were reported at IIER from January to November 2018, with 78 confirmed cases (69.0%). Of these, 23 individuals died (lethality of 29.5%). Males predominated among confirmed (80.8%) and discarded (74.3%) cases, without statistical difference between both groups (p=0.436). Analysis of age group among suspected population showed a predominance of adult cases (from 18 to 59 years old) (77.9%) and similar distribution between confirmed and discarded (p = 0.061). Whites were prevalent among suspect cases (69.9%), without statistical difference among confirmed and discarded (p = 0.183). Educational level was not different between those groups (p = 0.094), even though a larger proportion of individuals with up to 8 years of education or more is observed in the discarded group (91.4%) compared to the confirmed ones (78.4%). A higher proportion of individuals whose disease was discarded were previously vaccinated against YF (14.3%) compared to those who had the disease (3.8%), with no statistical difference (p = 0.105). Hospitalization occurred in 89.7% of confirmed cases, with significant difference (p < 0.001) if compared to the discarded cases (54.3%), as well as evolution to death, which occurred with 8.3% of those discarded and 29.5% of those confirmed, p = 0.016).

We analyzed signs, symptoms, and laboratorial alterations to verify possible predictive characteristics of YF among suspect cases attended. Initially, univariate analysis was performed, comparing confirmed and discarded cases of YF (Table 1). All variables with p < 0.20 were included in the logistic regression model. Two models show the significant and adjustment variables (Table 2).

**Table 1:** Signs, symptoms, and laboratorial alterations of suspect cases of yellow fever reported by Instituto de Infectologia Emílio Ribas. IIER, 2018.

Signs, symptoms, and laboratorial alterations	Total (n = 113)		Confirmed cases (n = 78)		Discarded cases (n = 35)		p^a^
		
	n	%	n	%	n	%	
Fever	111	98.2	77	100.0	31	91.2	0.027
Abdominal pain	109	96.5	45	58.4	16	50.0	0.419
Diarrhea	107	94.7	18	24.0	11	34.4	0.269
Hemorrhage signs	113	100.0	15	19.2	9	25.7	0.436
Renal excretion disorder^b^	111	98.2	24	30.8	2	6.1	0.005
Jaundice^b^	113	100.0	42	53.8	25	71.4	0.079
Myalgia	105	92.9	62	84.9	24	75.0	0.224
Headache	105	92.9	56	75.7	23	74.2	0.872
Chills	101	89.4	28	38.9	11	37.9	0.929
Nausea	105	92.9	58	77.3	23	76.7	0.941
Vomiting	105	92.9	52	69.3	18	60.0	0.359
Low back pain	98	86.7	36	51.4	20	71.4	0.071
Arthralgia	99	87.6	13	18.6	12	41.4	0.017
Thrombocytopenia	111	98.2	66	86.8	16	45.7	< 0.001
Leukopenia	111	98.2	47	61.8	6	17.1	< 0.001
Leukocytosis	111	98.2	5	6.6	5	14.3	0.281
ALT > 80U/L	111	98.2	76	100.0	22	62.8	< 0 .001
AST > 80U/L	111	98.2	75	98.7	22	62.8	< 0.001
Renal function alteration	111	98.2	36	47.4	12	34.3	0.196
Hyperbilirubinemia	112	99.1	37	48.1	19	54.3	0.541

ALT: alanine aminotransferase; AST: aspartate aminotransferase; 95%CI: 95% confidence interval; OR: odds ratio.

^a^Pearson qui-square test or Fisher’s exact test.

^b^Referred information.

**Table 2:** Associated factors to confirmed yellow fever in the Instituto de Infectologia Emílio Ribas. IIER, 2018.

Associated factors to yellow fever	OR unadjusted (95%CI)	p	OR adjusted (95%CI)	p	OR adjusted (95%CI)	p^a^
			
			Model 1		Model 2	
Abdominal pain	1.406 (0.614–3.218)	0.420				
Diarrhea	0.603 (0.245–1.485)	0.271				
Hemorrhage signs	0.688 (0.267–1.768)	0.437				
Renal excretion disorder^b^	6.889 (1.524–31.140)	0.012				
Jaundice^b^	0.4667 (0.198–1.100)	0.082				
Myalgia	1.878 (0.674–5.238)	0.228				
Headache	1.082 (0.412–2.837)	0.873				
Chills	1.041 (0.428–2.528)	0.929				
Nausea	1.038 (0.380–2.833)	0.941				
Vomiting	1.507 (0.625–3.633)	0.361				
Low back pain	0.423 (0.165–1.089)	0.075				
Arthralgia	0.323 (0.124–0.838)	0.020				
Thrombocytopenia	7.130 (2.83–17.91)	< 0.001	2.86 (0.96–8.54)	0.060	3.39 (1.11–10.32)	0.032
Leukopenia	7.590 (2.66–21.65)	< 0.001	4.26 (1.29–14.05)	0.017	5.88 (1.51–22.95)	0.011
Leukocytosis	0.420 (0.11–1.55)	0.190				
ALT > 80U/L	44.91 (5.56–362.57)	< 0.001			53.67 (5.26–547.36)	0.001
AST > 80U/L	22.16 (4.64–105.74)	< 0.001	14.27 (2.62–77.79)	0.002		
Renal function alteration	4.88 (1.06–22.54)	0.042				
Hyperbilirubinemia	0.851 (0.378–1.915)	0.697				

ALT: alanine aminotransferase; AST: aspartate aminotransferase; 95%CI: 95 % confidence interval; OR: odds ratio.

^a^Pearson qui-square test.

^b^Referred information.

**Figure 1:**
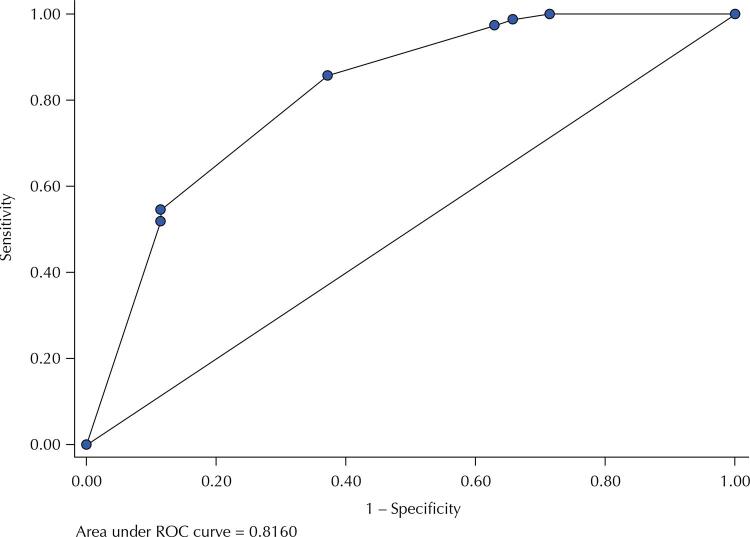
ROC curve for Model 1 (thrombocytopenia, leukopenia, and AST > 80), IIER, 2018.

**Figure 2:**
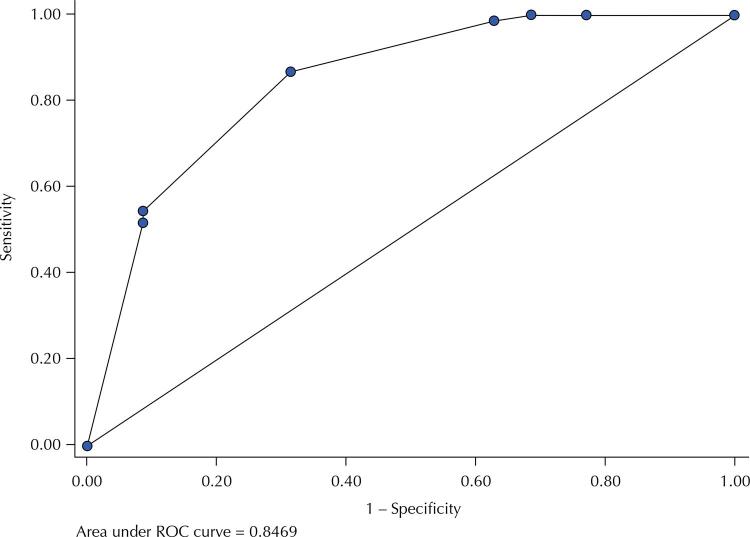
ROC curve for Model 2 (thrombocytopenia, leukopenia, and ALT > 80), IIER, 2018.

The quality of adjustment of the multivariate models were evaluated by Hosmer-Lemeshow test, whose p-value was not statistically significant (Model 1: p = 0.801; Model 2: p = 0.904), indicating good fit. In Model 1, significant predictive factors identified were leukopenia (OR= 4.26; 95%CI: 1.29–14.05) and the AST elevation superior to twice the reference value (OR= 14.27; 95%CI: 2.62–77.79) and thrombocytopenia (OR= 2.86; 95%CI: 0.96–8.54), p < 0.06. In Model 2, significant predictive factors were thrombocytopenia (OR= 3.39; 95%CI: 1.11–0.32), leukopenia (OR= 5.88; 95%CI: 1.51–22.95), and the ALT elevation superior to twice the reference value (OR= 53.67; 95%CI: 5.26–547.36). Figures 1 and 2 show the ROC curves for the Model 1 and Model 2:

Based on such factors, three different case definitions were proposed.

- Proposed definition 1: individual with acute onset of fever (reported or measured), followed by elevation of AST superior to twice the reference value AND leukopenia AND thrombocytopenia.
- Proposed definition 2: individual with acute onset of fever (reported or measured), followed by elevation of ALT superior to twice the reference value AND leukopenia AND thrombocytopenia.
- Proposed definition 3: individual with acute onset of fever (reported or measured), followed by elevation of ALT or AST superior to twice the reference value AND leukopenia OR thrombocytopenia.

Accuracy analysis was also compared with official definitions from Brazilian Ministry of Health (2017) and from World Health Organization (2015) (Table 3).

**Table 3:** Analysis of suspect case of yellow fever definitions according to quantitative attributes. IIER, 2018.

Definitions of suspect case	Sensitivity (%)	Specificity (%)	Predictive value positive-PVP (%)	Predictive value negative-PVN (%)	Accuracy (%)
Brazil’s definition	59.0	40.0	68.7	30.4	53.1
WHO’s definition	53.8	34.3	64.6	25.0	47.8
Proposed definition 1	54.5	88.6	91.3	47.0	65.2
Proposed definition 2	55.8	91.4	93.5	48.5	67.0
Proposed definition 3	88.3	62.9	84.0	71.0	80.4

IIER: Instituto de Infectologia Emílio Ribas.

Evaluation of sensitivity of suspect cases definitions indicates that only 59.0% met Brazil’s suspect case definition and 53.8% met WHO’s definition. Specificity was higher for Brazil’s definition (40.0%), while for WHO’s this indicator presented a lower value of 34.3%. PVP presented similar values, in both definitions, with higher index for Brazil’s definition (68.7%). PVN presented values of 30.4% and 25.0% in Brazil’s and WHO’s definitions, respectively. The definition with the most accuracy in the analyzed population was Brazil’s (53.1%), while WHO’s presented 47.8% accuracy in the analyzed population.

Proposed definition 1 presented low sensitivity (54.5%), high specificity (88.6%), high PVP (91.3%), low PVN (47.0%), and moderate accuracy (65.2%). Proposed definition 2 presented low sensitivity (55.8%), high specificity (91.4%), high PVP (93.5%), low PVN (48.5%), and moderate accuracy (67.0%). Proposed definition 3 presented high sensitivity (88.3%), moderate specificity (62.9%), high PVP (84.0%), moderate PVN (71.0%), and high accuracy (80.4%). Considering the criteria of all definitions described, proposed definition 3 was the most accurate.

## DISCUSSION

Case definition is an important tool for epidemiological surveillance, allowing detection of cases, estimation of incidence, and identification of epidemics. Standardization of case definition also contributes to evaluation of effectiveness of control measures and incidence comparison in different regions and periods. Case definition must be simple and useful, combining clinical, laboratorial, and epidemiological characteristics, depending on the monitoring objectives for each disease or health condition. A definition with high sensitivity and specificity is desirable, but these attributes must be balanced, considering the increase of sensitivity of great importance when extension of an epidemic needs to be assessed^10^. Balance must be reached between the need of high sensitivity at the expense of screening false-positive cases^11^.

Brazilian Ministry of Health and World Health Organization’s case definitions presented lower sensitivity, specificity, PVN, and accuracy, with moderate PVP. YF in Brazil presents low incidence, with significant increase of cases in 2017 and 2018, especially in the Southeastern region. A more sensitive case definition is necessary for diseases with low incidence, thus reducing the number of false negatives, considering the severity of disease and the possibility of prevention^12^.

Predictive factors of YF in the analyzed population were transaminases greater than twice the reference value, leukopenia, and thrombocytopenia. A study^13^ analyzed risk factors for death in 72 confirmed cases of YF and it identified, among recovered cases, the presence of leukopenia and increase of transaminases and thrombocytopenia. The analysis of regression showed independent factors related to death the increase of transaminases, age, and creatinine increase^13^. Another study, which analyzed 76 cases of YF, showed leukopenia, thrombocytopenia, and increase of transaminases among the cases. When risk factors for death were analyzed, there was statistical significance for the increase of transaminases, neutrophilia, and viral load^14^. Analysis of 52 YF confirmed cases showed increase of transaminases and thrombocytopenia. However, logistic regression showed that alteration of renal excretion and increase of transaminases were significantly associated to death^15^. A study analyzing confirmed cases of YF attended in ICU identified factors associated to death, serum lipase and, as protection, factor V. Nonetheless, in the univariate analysis there was increase of transaminases, bilirubin, lipase, INR and lactate in patients who died, and no alteration of viral load when compared to cases which evolved to cure^16^.

Proposed case definition 2 presented moderate (67,6%), sensitivity of 56,6% specificity of 91.4%, PVP of 93.5%. This case definition includes fever, increase of transaminases (AST superior to twice), and thrombocytopenia or leukopenia. The exams are simple, inexpensive, and improve sensitivity for detection of cases. Proposed definition 1 presents accuracy of 65.8%, sensitivity of 55.3%, and specificity of 88.6%, PVP 91.3%. In this proposed definition, the presence of fever was followed by laboratorial alterations, increase of transaminases (ALT superior to twice), leukopenia, thrombocytopenia. This proposed definition has lower sensitivity and higher specificity. Proposed definition 3 presented high sensitivity (88.3%), moderate specificity (62.9%), high PVP (84.0%), moderate PVN (71.0%), and high accuracy (80.4%). In this proposed definition, the presence of fever was followed by elevation of ALT or AST greater than twice the reference value and leukopenia or thrombocytopenia. Considering the epidemiological situation with high or low case incidence, we must adapt the case definition regarding sensitivity and specificity. In a scenario of low incidence of disease, a higher sensitivity is desirable, even if it increases the number of false positives, to minimize the number of non-detected cases. However, a surveillance system with high PVP, low false positive reports, would not lead to wasted resources on cases that do not actually exist^11^. Therefore, definitions proposed by Brazilian Ministry of Health and WHO presented low sensitivity, thus requiring more sensitive definitions to detect most of YF cases. Other studies developed definitions with the use of similar methodology^17^. Incorporation of laboratorial results made the definition more sensitive, since transaminases alterations in serum are earlier identified than clinical manifestation of jaundice, a signal included in both case definitions (Brazil and WHO) that may be absent in some cases^18^.

Limitation of study may be attributed to the use of data from one hospital only, consequently with a small sample size that can lead to inaccuracies in the association measures. However, Instituto de Infectologia Emílio Ribas is a reference public hospital for infectious disease in the state of São Paulo. Furthermore, cases were identified during a YF epidemic in some regions of the state. Thus, increasing the chance of virus detection in investigated cases. Most cases confirmed the YF virus detection by real-time polymerase chain reaction (RT-PCR) technique (91%). Only seven cases were confirmed by serology, an exam that can cross-react with other flaviviruses. However, four of these cases had jaundice, one associated with hemorrhage that progressed to death. These cases were from regions with yellow fever transmission in the same period. The use of laboratorial exams to case definition can be difficult in regions with lack of access to laboratory diagnosis. Nevertheless, the inclusion of laboratorial criteria, associated to symptomatology, increased sensitivity of case definition, making it useful in situations of outbreak and in sentinel investigation of feverish syndromes for differential diagnosis, especially considering coexistence of many arboviruses in Brazil with similar symptomatology. Furthermore, detection of YF virus in densely populated peri-urban regions with a high rate of *Aedes aegypti* infestation increases the risk of urban YF epidemic. Therefore, case definitions with high accuracy allow implementation of more timely preventive measures, such as vaccination of population under risk.
